# Routine 64-slice spiral CT scanning in diagnosis of Thoracic trauma

**DOI:** 10.1186/1471-227X-12-S1-A3

**Published:** 2012-12-18

**Authors:** MENG Fanshan, LIU Debin, CUI Xuefeng

**Affiliations:** 1 Emergency Department, the 88th Hospital of PLA, Taian 271000, Shandong, China

## Objective

To investigate the value of routine 64-slice spiral CT scanning in diagnosis for patients with acute thoracic trauma in emergency room.

## Methods

The clinical data of 268 patients with chest wound who were admitted to our hospital in recent years after 64-slice spiral CT scanning and X-ray check were reviewed and analyzed.

## Results

Among these patients with chest wound 206 patients were found distinct injury after CT inspection, but only 108 patients were found injured with X-ray check. The CT scanning results were as follows: lungs were damaged in 148 cases, including 87 with lung contusion, 52 with lacerated wound, and 9 with tracheal bronchial tube damage. Damages outside the lungs were seen in 126 patients, including 56 patients with trauma in pleural membrane (22 cases of hemothorax, 26 cases of pneumothorax and 8 cases of blood pneumothorax), 9 cases with mediastinum damage (5 cases of vertical mediastinal pneumatosis, 4 cases of hematocete), 78 cases with thoracic wall damage (59 cases of rib bone fracture, 19 cases of breastbone fracture).

## Conclusion

CT scanning is a principal way in the diagnosis of chest wound. It has the advantages of quick scanning and the characteristics of high sensitivity, and it will certainly play a vital role in first-aid process in emergency medical treatment of chest wound.

